# B chromosome and its non-Mendelian inheritance in *Atractylodes lancea*

**DOI:** 10.1371/journal.pone.0308881

**Published:** 2024-09-11

**Authors:** Kazuya Hara, Shinji Kikuchi, Misaki Inoue, Takahiro Tsusaka, Miki Sakurai, Hideyuki Tanabe, Kenta Shirasawa, Sachiko Isobe

**Affiliations:** 1 Graduate School of Horticulture, Chiba University, Chiba, Japan; 2 Plant Molecular Science Center, Chiba University, Chiba, Japan; 3 Research Center for Space Agriculture and Horticulture, Chiba University, Chiba, Japan; 4 Botanical Raw Materials Division, Tsumura & Co., Ibaraki, Japan; 5 Research Center for Integrative Evolutionary Science, SOKENDAI, Kanagawa, Japan; 6 Kazusa DNA Research Institute, Chiba, Japan; Tulane University Health Sciences Center, UNITED STATES OF AMERICA

## Abstract

Supernumerary B chromosomes contribute to intraspecific karyotypic variation. B chromosomes have been detected in more than 2000 organisms; they possess unique and diverse features, including non-Mendelian inheritance. Here, we report one or more B chromosomes in the gynodioecious plant *Atractylodes lancea*. Among 54 *A*. *lancea* lines, 0–2 B chromosomes were detected in both hermaphroditic and female plants, with the B chromosomes appearing as DAPI-bright regions within the nuclei. Genomic *in situ* hybridization revealed that the B chromosomes had no conserved A chromosome DNA sequences, confirmed by fluorescence *in situ* hybridization probed with independently dissected B chromosomes. In male meiosis, the B chromosome did not pair with an A chromosome and was therefore eliminated; accordingly, only 20.1% and 18.6% of these univalent B chromosomes remained at the end of meiosis for the 1B lines of KY17-148 and KY17-118, respectively. However, we also found that B chromosomes were transmitted from male parents in 40.8%–44.2% and 47.2% of the next generation; although these transmission rates from male parents were not essentially different from Mendelian inheritance (0.5), the transmission of gametes carrying B chromosomes increased through fertilization or seed development. B chromosomes were transmitted from three of four 1B female parents to 64.3%–92.6% of the next generation, suggesting B chromosome accumulation. We propose that the B chromosome of *A*. *lancea* has a specific sequence and persists via non-Mendelian inheritance from female parents. Overall, *A*. *lancea*, with its unique characteristics, is a promising model for understanding the structure, evolution, and mechanism of non-Mendelian inheritance of B chromosomes.

## Introduction

Although each species possesses a particular karyotype, karyotypic changes are occasionally detected within many species. These changes can occur due to polyploidy, aneuploidy, and chromosomal rearrangement events such as chromosomal fusion, fission, and translocation. Moreover, heteromorphic sex chromosomes and XO and ZO sex-determination systems can cause different karyotypes within a species.

Since the discovery of supernumerary chromosomes in Hemiptera by Wilson [1], B chromosomes (Bs)—extra dispensable chromosomes—have been detected in more than 2000 plant and animal species [2–5]. Supernumerary B chromosomes were named in contrast to the standard A chromosomes [6], and they also cause karyotypic changes. Because B chromosomes are not essential, individuals with multiple copies (in some cases >10) of the B chromosome have been identified within a species [7, 8]. In some cases, such accessory chromosomes are maintained within a population by non-Mendelian inheritance [9]. The phenomenon of a chromosome being transmitted to progeny at a rate more significant than the Mendelian rate (0.5) is known as genetic drive, and this appears in several species as a feature of B chromosomes (Bs); however, Bs lacking genetic drive are also known to exist [10]. The genetic drive can take several forms, including nondisjunction, mitotic drive (e.g., in *Crepis capillaris*; [10]), meiotic drive, and transmission drive (or preferential fertilization), before, during, and after meiosis [11]. The frequency of B chromosomes within a population is determined by the host’s fitness and the drive mechanism’s efficiency [4].

The drive mechanism of B chromosomes has been thoroughly investigated in rye and maize. In rye, B chromosomes are transmitted into the generative nucleus due to the failure of chromatids to separate during cell division, i.e., via nondisjunction by the heterochromatic end of the long arm and asymmetric spindle formation during pollen (male) mitosis I [3, 12–14]. Interestingly, nondisjunction of rye B chromosomes has also been observed during the first mitosis within the embryo sac (female) [3, 15]. In maize, two dive mechanisms have been reported as linked to the formation of B chromosomes in maize [16]: (i) nondisjunction during the second pollen mitosis in the generative nucleus [17–19] and (ii) preferential fertilization of the egg cell rather than the central cell by sperm containing two B chromosomes [20]. Beyond rye and maize, in *Lilium callosum*, a female meiotic drive has been detected, wherein the B chromosome passes toward the micropylar side at a frequency of 63.7% [21]. The number of B chromosomes may also differ among organs, for example, due to programmed elimination in the roots during embryo development in *Aegilops speltoides* [22].

What are the general structural features of B chromosomes? Although B chromosomes accumulate multiple mutations and structural changes, they are believed to have originated from the A chromosomes of the same species or from a relic of a cross such that Bs were introgressed via interspecific cross, after which they persisted and evolved (reviewed by [3]). In plants, B chromosomes are generally smaller than A chromosomes. Structural polymorphisms of B chromosomes, such as large B and micro B, have been reported in the daisy *Brachycome* [23, 24]. Plant B chromosomes may be euchromatic [10] but are often heterochromatic. This heterochromatic nature derives from accumulating several specific repetitive sequences, such as rye D1100, E3900, and ScCI11 [14]. The structural and epigenetic specificity of the B chromosome suppresses its ability to pair with an A chromosome during meiosis, a behavior characteristic of the B chromosomes. Recently, a high-quality sequence of the maize B chromosome was released [25], in which the authors predicted 758 protein-encoding genes (including at least 88 expressed genes) present in 125.9 Mbp of the B chromosome sequence; the absence of synteny among predicted B genes suggested that it evolved via translocation from the A chromosome [25]. Moreover, genes on the independently evolved B chromosome affected gene expression on the A chromosome [26, 27]. Traits are also present on B chromosomes in other species: for example, the rye B chromosome confers heat tolerance during microsporogenesis [28], and the *Allium* B chromosome promotes rapid seed germination [29, 30].

The gynodioecious species *Atractylodes lancea* De Candolle, in the family Asteraceae (Compositae), is distributed in East Asia [31], and its dried rhizomes have been used as crude drugs, generally to treat digestive disorders and body fluid imbalances, in Chinese and Japanese traditional herbal medicines [32, 33]. Its chromosome number is 2*n* = 24 [34, 35], and genome sequencing, genetic marker development for medicinal compound identification, genetic analysis, and molecular breeding research projects are underway based on this chromosome number [36–38]. However, in this study, we report the identification of a novel extra chromosome in *A*. *lancea*. Our cytological observations revealed that extra chromosomes are widely present in the species. We, therefore, sought to determine in this study (i) whether this excess chromosome is responsible for karyotypic variation in *A*. *lancea*, (ii) whether this excess chromosome can be considered a B chromosome insofar as it shows characteristic B chromosome structural features and behaviors, and (iii) whether this extra chromosome causes genetic drive. Finally, we propose *A*. *lancea* as a new model system for research to understand the structure, evolution, and mechanism of non-Mendelian inheritance mechanisms of B chromosomes.

## Materials and methods

### Plant materials

*A*. *lancea* De Candolle was grown in an experimental field at Tsumura and Co., Japan, in 2020, 2021, and 2023. We used 54 resource lines (see **S1 Table**) of *A*. *lancea*. Each originated from China and was preserved by Tsumura and Co. [37]. Several plants that generated F_1_ seedlings were prepared in 15 cm pots in a growth chamber with a fixed temperature of 28°C.

### Chromosome preparation

Mitotic chromosomes were obtained from elongated fresh roots of plants grown in 15 cm pots. After excision, roots were treated with 2 mM 8-hydroxyquinoline at 18°C for 6 h, then fixed with 3:1 (v/v) ethanol–acetic acid at 18°C for 5 days. Next, fixed roots were stored in 70% ethanol at 4°C until use. Meiotic chromosomes were obtained from young anthers (i.e., 1.0–1.5 mm in length). Excised anthers were fixed using the same procedure used for the root samples.

Next, mitotic and meiotic chromosome slides were prepared using the enzymatic maceration–squash method [39]. The chromosomes were counterstained with 5 μg/mL of 4,6-diamidino-2-phenylindole (DAPI) in VECTASHIELD (Vector Laboratories, USA) and then observed under a BX43 phase-contrast microscope or a BX-53 fluorescence microscope (both from Olympus, Japan). All fluorescence images were captured using a CoolSNAP MYO CCD camera (Photometrics, USA), and the resulting images were processed with MetaVue/MetaMorph version 7.8 (Molecular Devices, Japan), Adobe Photoshop CS3 v10.0.1 (Adobe, Japan), and NIH ImageJ (https://imagej.nih.gov/ij/). Finally, all chromosomes were counted in at least 10 mitotic cells per line.

### Genomic *in situ* hybridization (GISH) and fluorescence *in situ* hybridization (FISH) analyses of mitotic and meiotic chromosomes

Total genomic DNA was extracted from the young leaves of three lines, 5-7-32 (2*n* = 24), YB2019-30 (2*n* = 25), and YB2019-3 (2*n* = 26), using a DNAs-ici!-R DNA extraction kit (Rizo Inc., Tsukuba, Japan). Extracted DNA was then stored at -30°C until further use. GISH probes were prepared from DNA samples using a Biotin-Nick Translation Mix or a DIG-Nick Translation Mix (both from Sigma, USA). A pTa71 plasmid [40] was also used for DIG-Nick labeling for the rDNA probe. The 2*n* = 24 probe and rDNA probe were detected using DIG–rhodamine, and the 2*n* = 25 and 2*n* = 26 probes were detected using Biotin–streptavidin FITC. All GISH and FISH analyses were conducted using a previously described protocol [41].

### Microdissection and preparation of FISH probes using dissected DNA

Microdissection was performed according to a modified protocol described in a previous report [42]. Microdissection chromosome slides prepared from Y-T16-69 (2B) were first stained with Giemsa solution. Fine glass needles were prepared using a puller (NARISHIGE PC-10) and sterilized using a UV irradiation system (CL-1000, UVP, FUNAKOSHI). Microdissection was performed using a glass needle attached to a micromanipulator (Eppendorf TransferMan NK 2) under a light microscope (OLYMPUS IX71). Specifically, a single chromosome was microdissected, scraped, and then transferred to a PCR tube. The dissected single chromosome—we used one chromosome (i.e., ~0.37 pg) per tube—was subjected to amplification using a PicoPLEX® Gold Single Cell DNA-Seq Kit (Takara Bio) and indexing primers (DNA HT Dual Index kit, Takara Bio) with all procedures performed as per the manufacturer’s protocol. Four replicates (i.e., #9, #8, #5, and #2) were obtained by repeating the above operation. Next, four DNA probes were prepared using the “Library Amplification” protocol of the PicoPLEX® Gold Single Cell DNA-Seq Kit (50 μl of total volume per reaction; 12 amplification reaction cycles) using 1 μl of 1 mM digoxigenin-11-dUTP (Roche) and 1 μl of purified library + 19 μl of Milli-Q water instead of 20 μl preamplification cleanup DNA. These four DNA probes were then used as FISH probes. FISH was performed as per a previously described protocol [41].

### Estimation of pollen fertility

Mature pollen grains were stained with acetocarmine and classified under a BX43 microscope as fertile (i.e., strong and uniform staining) or sterile (i.e., poor or no staining). Pollen was collected from at least three flowers of KY17-60 (2*n* = 24), KY17-93 (2*n* = 24), KY17-10 (2*n* = 25), KY17-15 (2*n* = 25), and YB2019-3 (2*n* = 26), and more than 300 pollen grains per line were scored.

### Generation of F_1_ seedlings

Ten female lines (i.e., KY17-5, KY17-6, KY17-7, KY17-21, KY17-29, KY17-30, KY17-38, KY17-43, KY17-45, and Y-T16-69) and seven hermaphroditic lines (i.e., KY17-15, KY17-22, KY17-37, KY17-60, KY17-118, KY17-148, and YB2019-3) were used as parents to produce F_1_ seedlings. Rhizomes of each strain were first divided into 30 g pieces and planted in 15 cm pots in December 2020. Plantlets were grown under natural conditions in Ami-machi, Inashiki-gun, Ibaraki Prefecture (35° 99′ N, 140° 20′ E), Japan, until August 2021. After that, all plants were moved to a growth chamber and grown for 4 weeks under 16 h light at 25°C/8 h dark at 20°C. During the next 24 days, crosses were performed between the strains with mature pistils and stamens. The cross combinations used are described in detail in subsequent paragraphs. After artificial pollination, plants were grown under the same conditions for another four weeks under 12 h light at 20°C/12 h dark at 15°C and under 12 h light at 15°C/12 h dark at 5°C for a further two weeks. After this point, the F_1_ seeds were harvested, sown, and grown in a greenhouse at 25°C.

### Statistical analysis of transmission rates

We calculated the transmission ratio (*k*_*B*_)—i.e., the mean number of Bs in the microspores or progeny divided by the total number of Bs in the parents—and Z values for parents with B chromosome(s) using a formula described in a previous report [43]. The data were used to determine the accumulation of Bs by comparing *k*_*B*_ with the expected Mendelian rate (0.5). We did not collect data for “embryos,” as in previous studies of other species [43], due to technical difficulties related to embryo observation in *A*. *lancea*. Instead, we replaced the number of embryos with the number of seeds. Thus, in this study, the transmission rates to the next generation are values obtained by compiling meiosis, gametogenesis, reproduction, and seed development. The Z-values indicate significant B accumulation when >1.96 in absolute value and significant B elimination when Z values <–1.96 [43].

## Results

### Novel extra chromosome(s) in *A*. *lancea*

**Fig 1:**
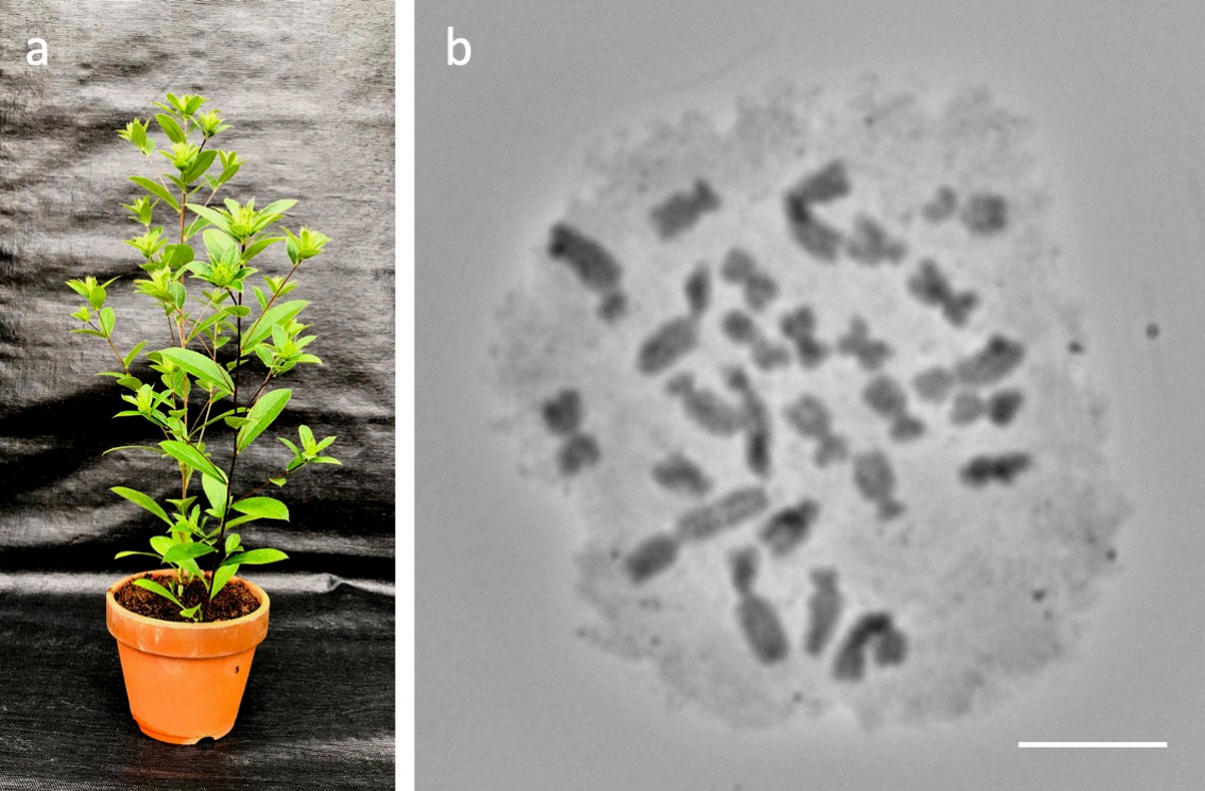
A clone of the *Atractylodes lancea* line Y-T16-69. (a) Morphology typical of the species. (b) Mitotic chromosomes from a root tip (2*n* = 26). Scale bar = 10 μm.

**Fig 2:**
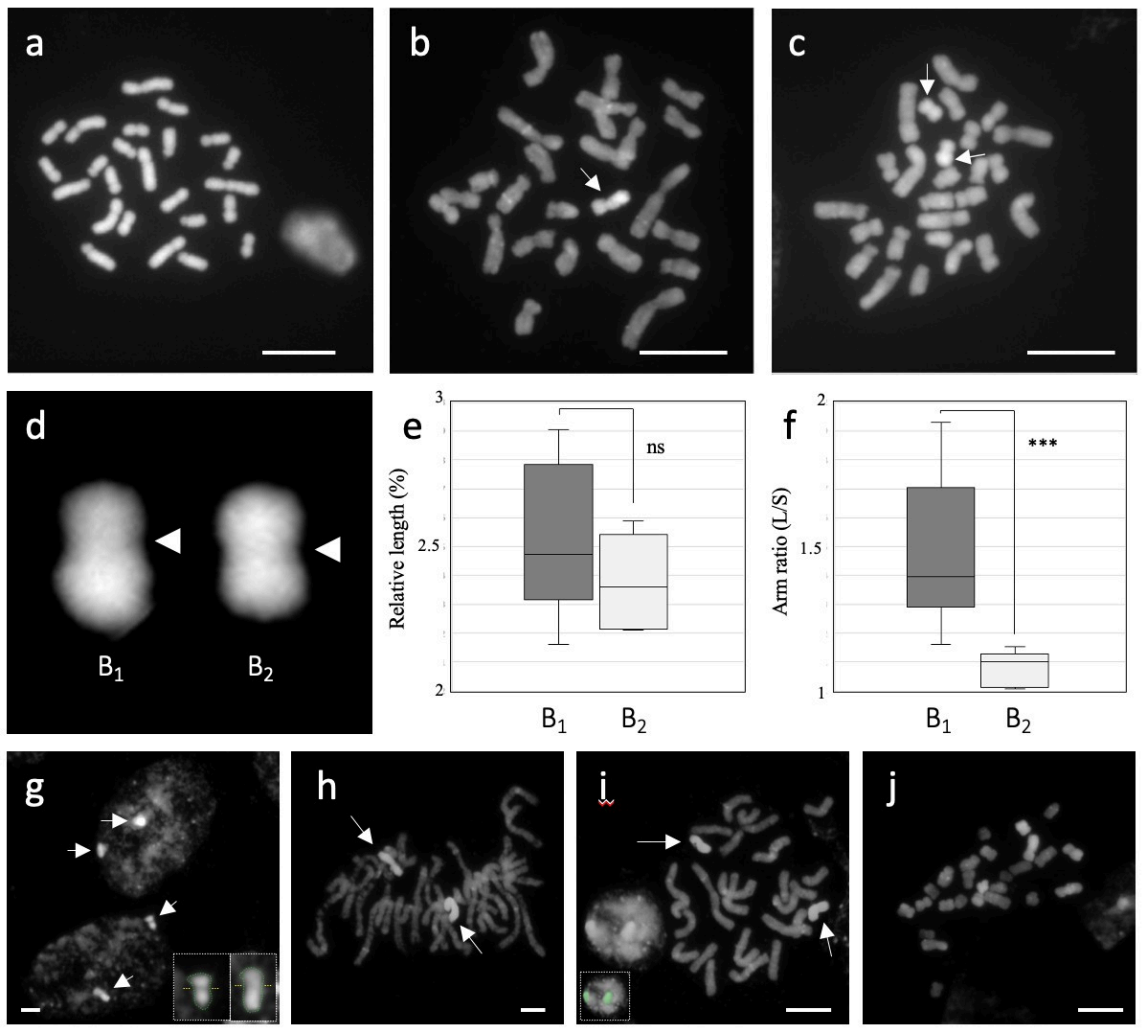
B chromosomes in *A*. *lancea*. (a) KY17-43 showed 2*n* = 24. (b) KY17-6 (2*n* = 25) showed 24 chromosomes and a DAPI-bright B chromosome (arrow). (c) Y-T16-69 (2*n* = 26) had 24 and two B chromosomes (arrows). (d) Cut-out images of two types of B chromosomes. B_1_, which has distinguishable short and long arms, and metacentric-like B_2_. Arrowheads indicate centromere positions. (e) Y-axes present the relative length (%) of the B chromosome to all 26 chromosomes (i.e., length of the B chromosome (mm) / length of all 26 chromosomes (μm) × 100). No significant differences were detected in the relative length of the two Bs (Student’s *t* test; *P* > 0.1). (f) Y-axes represent arm ratio (L/S: long arm / short arm). A statistically significant difference (*P* < 0.01) was detected in the arm ratios of B_1_ and B_2_. (g–i) Bs (arrows) of Y-T16-69 during the cell cycle. (g) Interphase. DAPI-bright regions of the two Bs can be observed. Note: The size of the DAPI-bright region is comparable to that of the B chromosome, and primary constriction can often also be observed. Inset: Green and yellow dot lines indicate the shape of the B chromosome in interphase and primary constriction sites, respectively. (h) Prometaphase. Bs have completed chromatin condensation. (i) Prometaphase. Due to the chromatin condensation of A chromosomes being almost complete, Bs become less visible. Note: The nucleus on the left is more condensed than the nuclei in Fig 2G, and DAPI signals can be observed that indicate the shape of the two B chromosomes (see green colored boxes in the inset). (j) Metaphase. Completely condensed chromatin due to anti-tubulin treatment with 2 mM 8-hydroxyquinoline. Further chromosome condensation has made the B chromosomes even less distinguishable from the A chromosomes. Scale bars = 10 μm.

Line Y-T16-69 showed typical *A*. *lancea* morphology (**Fig 1A**). However, its chromosome number was 2*n* = 26 (**Fig 1B**), whereas *A*. *lancea* reportedly has 2*n* = 24 [34, 35]. Moreover, the number of B chromosomes was stable, with no variation between the somatic cells of root tips (**Fig 1B**) and anthers. Therefore, we counted the chromosomes of the 54 lines of *A*. *lancea* (**S1 Table**) and detected three karyotypes, viz., 2*n* = 24, 2*n* = 25, 2*n* = 26 (**Fig 2A–2C** and **Table 1**). Bright DAPI-stained chromosomes were detected in addition to the normal karyotype 2*n* = 24 (**Fig 2A–2C**). Thus, 2*n* = 24 is a normal karyotype, while 2*n* = 25 and 2*n* = 26 represent genotypes with 1 and 2 extra chromosomes, respectively. We detected the extra chromosomes in both hermaphroditic and female plants (**Tables 1** and **S1**), suggesting a lack of contribution to sex determination. Hereafter, the extra chromosome is referred to as the B chromosome.

**Table 1:** Chromosome numbers in 54 lines of *A*. *lancea*.

	Obs.	Chromosome number		
		2*n* = 24	2*n* = 25	2*n* = 26
Female plant	28	15	11	2
Hermaphroditic plant	26	18	7	1
Total (%)	54	33 (61)	18 (33)	3 (6)

### Two distinct morphologies of B chromosomes

We observed that the two B chromosomes in line Y-T16-69 had different morphologies (**Fig 2D**). They showed similar lengths to the total length of all 26 chromosomes (average relative length = 2.5% ± 0.4 and 2.3% ± 0.2, n = 10) but had different arm ratios (**Fig 2E and 2F**). The submetacentric-like B chromosome (average arm ratio = 1.5 ± 0.2, n = 10) was named B_1_, and the metacentric-like B chromosome (average arm ratio = 1.1 ± 0.1, n = 10) was named B_2_ (**Fig 2D–2F**). A difference in the arm ratio was also confirmed from the chromosome images stained with Giemsa (**S1 Appendix**).

### B chromosomes in the cell cycle

Next, DAPI staining revealed that the B chromatin was a DAPI-bright region during the cell cycle (**Fig 2G–2J**); moreover, the primary constriction and shape of the B chromosome were visible in the nucleus (**Fig 2G and 2I**). However, identifying Bs was difficult in the metaphase since other A chromosomes became condensed to the same level (**Fig 2J**).

### Specific DNA of B chromosomes

**Fig 3:**
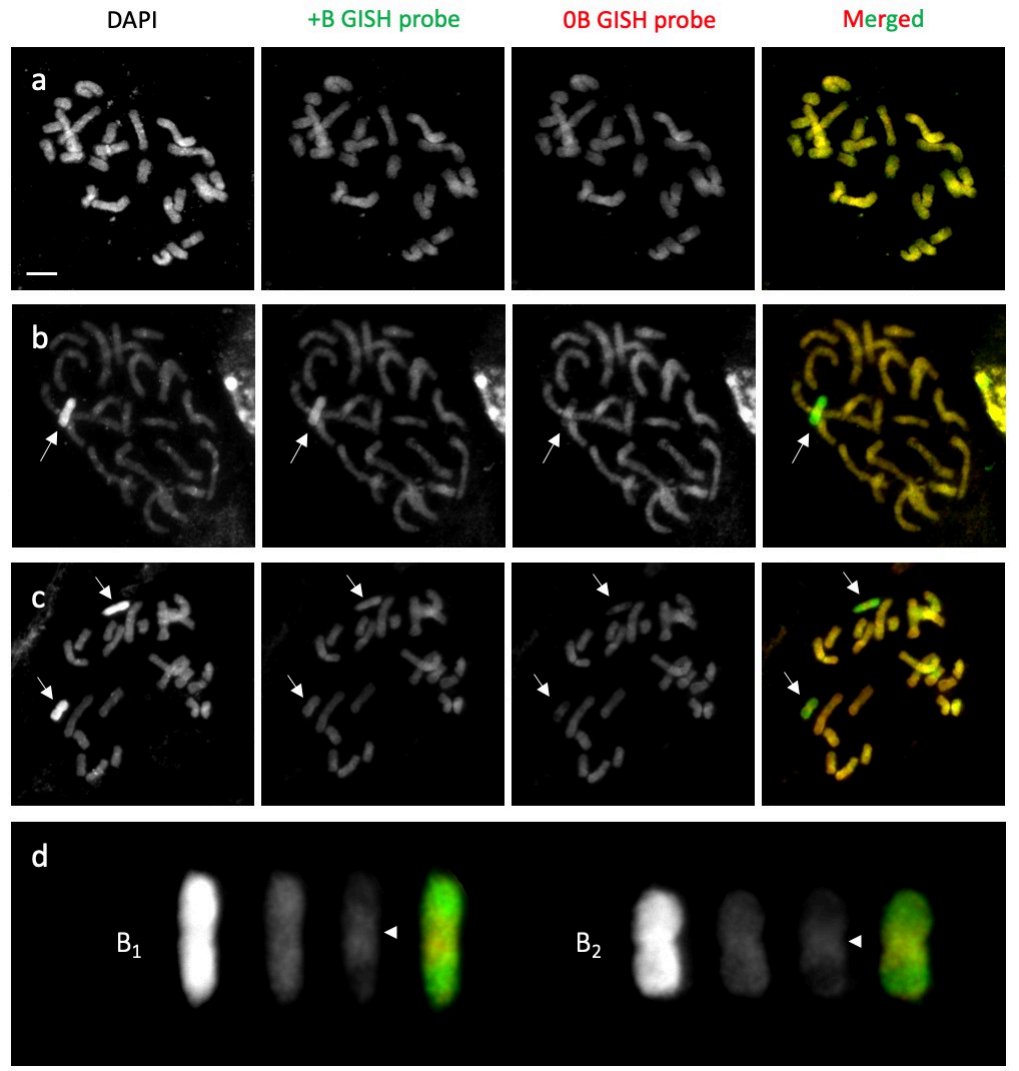
GISH analysis with the gDNA probe (red) for 2*n* = 24 (0B) and gDNA probe (green) for 2*n* = 25 (+1B) or 2*n* = 26 (+2B). (a) 5-7-32 (2*n* = 24). The 24 chromosomes are indicated in yellow in the merged image here and in (b) and (c). (b) YB2019-30 (2*n* = 25). The B chromosome (arrow) is indicated in green. (c) YB2019-3 (2*n* = 26). GISH probe derived from 2*n* = 26 gDNA (+2B GISH probe) showing green fluorescence signals on both Bs (arrows). (d) Cut-out images of the Bs are shown in panel (c). The centromere regions are hybridized with the 0B probe (arrowheads). Scale bar = 10 μm.

We conducted GISH analysis using labeled genomic DNA as probes to determine whether B chromosomes had DNA sequences conserved with the A chromosomes. For this procedure, 2*n* = 24 (0B) was detected as red, and 2*n* = 25 (+1B) or 2*n* = 26 (+2B) was detected as green (**Fig 3A–3C**). Moreover, all A chromosomes were yellow (**Fig 3A**). Our results showed that almost all B chromosomes were green (**Fig 3B and 3C**), except for the yellow centromeric regions labeled with 0B and the +1B or +2B GISH probes (**Fig 3D**). We interpreted this result as GISH analysis revealing that B chromosomes accumulate specific DNA sequences, with only the centromeric region having sequences similar to A chromosomes. FISH analysis was performed using 35S rDNA as a probe (labeled pTa71 clone, typically visualized as NORs) produced two pairs of four FISH signals (which is consistent with a previous report [35]) and showed no signal on the B chromosomes (**S2 Appendix**).

### Meiotic behavior of B chromosomes

**Fig 4:**
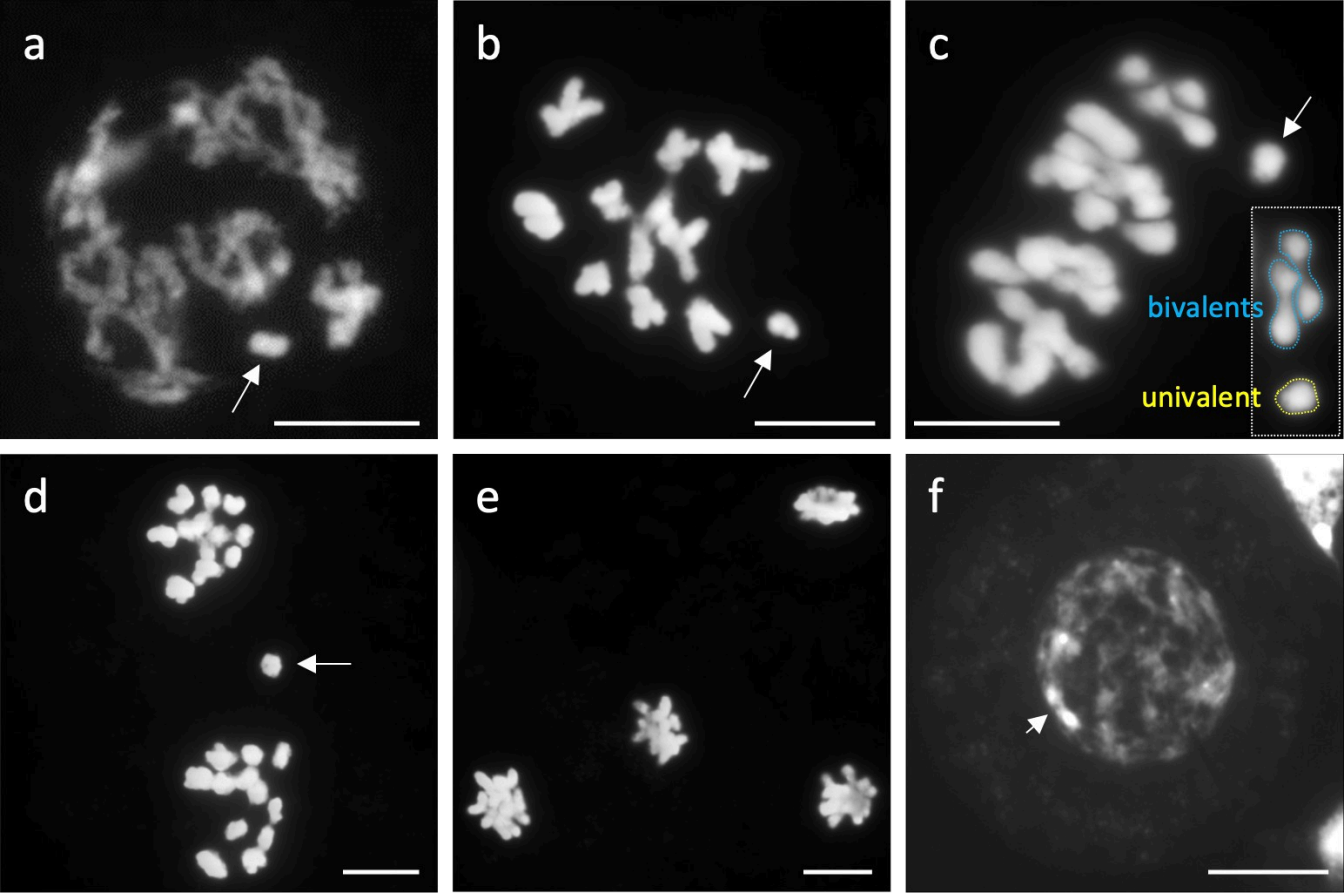
Meiosis in *A*. *lancea* KY17-15 (2*n* = 25). (a) Diplotene. The condensed B chromosome (arrow) does not pair with other A chromosomes. (b) and (c) Diakinesis and metaphase I. Arrows indicate univalent chromosomes. (d) Telophase I. Arrow indicates a lagging chromosome. (e) Telophase II. No lagging chromosomes or micronuclei are observable. (f) A microspore. The arrow indicates a DAPI-bright region of a B chromosome. Scale bars = 10 μm.

Next, we observed the behavior of B chromosomes during male meiosis (**Fig 4**). In a 0B plant, 12 bivalents were formed, and regular meiosis was observed without univalent or lagging chromosomes. However, in a 1B plant (KY17-15), a univalent or lagging chromosome appeared from diplotene to anaphase I (**Fig 4A–4D and S3 Appendix**). Moreover, since the univalent chromosome can be recognized as a condensed B chromosome in the diplotene stage (**Fig 4A**), we considered the B chromosome as a lagging chromosome. Such abnormal chromosomes did not appear during the second division of meiosis (**Fig 4E**).

### Transition of B chromosomes to microspores

One B chromosome in the 1B plant formed a univalent in male meiosis (**Fig 4B and 4C**). Under the non-drive hypothesis, chromatid segregation transmits the B to two of the four daughter cells, i.e., with a frequency of 50%. We examined the transmission rate of B of *A*. *lancea* to daughter cells (microspores).

**Fig 5:**
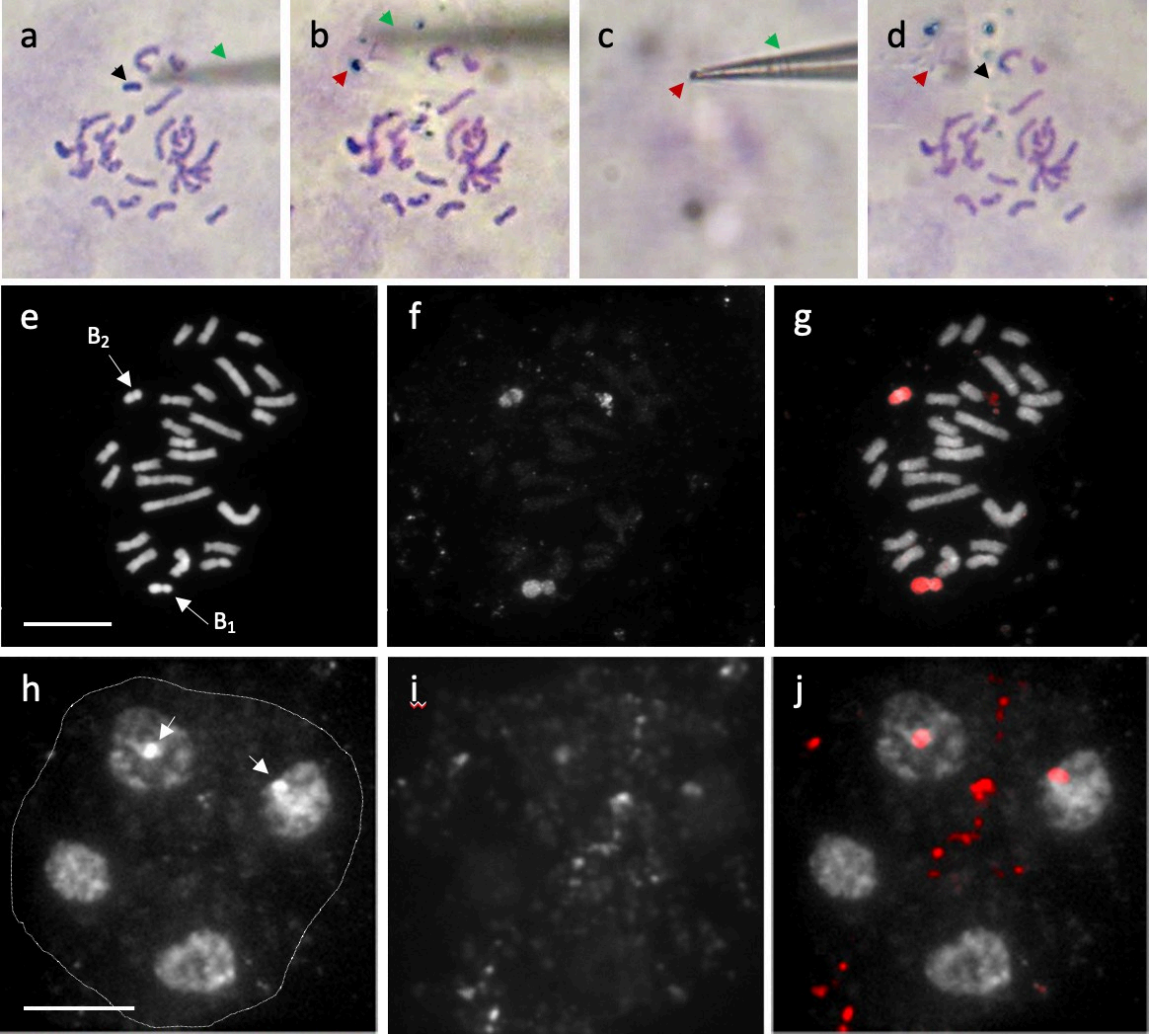
Microdissection of an *A*. *lanceae* B chromosome. (a–d) Microdissection of an *A*. *lanceae* B chromosome. (a) A fine glass needle (green arrow) approaches one of the B chromosomes (black arrow). (b) The glass needle (green arrow) scrapes off the target B chromosome. The red arrow indicates a shaved B chromosome. (c) The shaved B chromosome (red arrow) was taken with the tip of a glass needle (green arrow). (d) The spread chromosome following microdissection. Note: Nothing else is present where the target B chromosome (black arrow) and the B chromosome that was scraped off (red arrow) were located. (e–g) FISH probed with microdissected DNA (probe #9). (e) DAPI-stained chromosome spread of Y-T16-69 carrying B_1_ and B_2_. (f) FISH signals from the dissected DNA probe. (g) Merged image. The dissected DNA probe formed FISH signals on both B chromosomes. (h–j) FISH analysis of a tetrad. (h) DAPI-stained tetrad with four nuclei. A line indicates the outline of the tetrad. DAPI-bright regions (arrows) are observed in two of the four nuclei generated by meiosis. (i) FISH signals from the dissected DNA probe (probe #9). (j) Merged image. DAPI-bright regions overlapped with the B-specific probe. Scale bar = 10 μm.

The nuclei of microspores (i.e., the daughter cells produced after male meiosis) showed either no or only one DAPI-bright signal derived from the B chromosome in 1B plants (**Fig 4F and S4 Appendix**). To confirm that the DAPI-bright region is derived from the B chromosome, we developed B-specific probes by microdissection (**Fig 5**). 11 B chromosomes were excised without distinguishing between B_1_ and B_2_ (**Fig 5A–5D**), and the dissected DNAs were amplified for each. For 4 (probes #9, #8, #5, and #2) of the 11 amplified dissected DNAs, FISH analyses of mitotic chromosomes in a 2B line (Y-T16-69) were conducted without any blocking DNAs (**Fig 5E–5G and S5 Appendix**). The results indicated that all four probes formed signals on both B chromosomes, suggesting the successful development of the B-specific probes (**Fig 5E–5G and S5 Appendix**).

**Fig 6:**
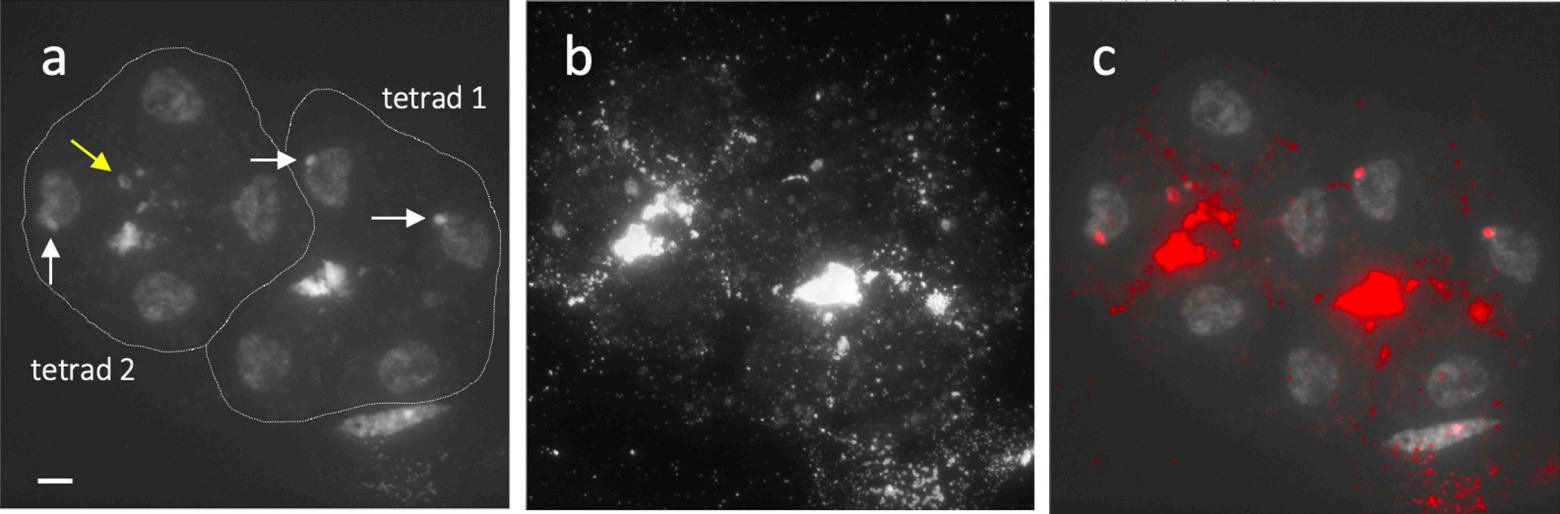
Micronucleus with B-DNAs during male meiosis. (a) Two DAPI-stained tetrads after male meiosis. (b) FISH signal for B-probe #9. (c) Merged image. White arrows and lines indicate DAPI-bright regions with B-DNA and the outline of the tetrad, respectively. The right tetrad (tetrad 1) shows B segregation into two nuclei. The left tetrad (tetrad 2) has only one nucleus carrying B-DNAs and a micronucleus with B-DNAs (yellow arrow). Scale bar = 10 μm.

Next, using the B-specific probes, we confirmed the B origin of the DAPI-bright region in the nuclei of the tetrad after male meiosis. As a result, the DAPI-bright signals overlapped with B signals (**Fig 5H–5J**), indicating that the DAPI-bright region could be counted as the number of B chromosomes. Moreover, the FISH analysis revealed the appearance of micronuclei with B-DNAs (**Fig 6**). In diploids, a single A chromosome is transmitted to the nucleus of each daughter cell by reduction division. We did not observe two DAPI-bright regions per nucleus of microspores of 1B line, which would suggest a meiotic drive (nondisjunction). Because we observed the micronuclei containing B-DNA in the tetrad, we suspected that the B chromosomes were transmitted lower than the Mendelian ratio. Therefore, we investigated the meiotic transmission rate of univalent B chromosomes to microspores using two 1B lines by counting the DAPI-bright region and found that the B chromosome was transmitted to 18.6%–20.1% of the microspores (**Table 2**). In contrast, the predicted Mendelian rate was 50%.

**Table 2:** Transmission rate of one B chromosome to microspore.

	Obs. ^1)^	No. of microspore (%)		*kB*	Z ^2)^
		0B	1B		
KY17-148 (1B)	675	539 (79.9)	136 (20.1)	0.201	**-15.511**
KY17-118 (1B)	698	568 (81.4)	130 (18.6)	0.186	**-16.579**

^1)^ Obs. = the number of observed microspores.

^2)^ B elimination (Z values lower than -1.96) were indicated by underline.

### Fertility of pollen containing B chromosome

The pollen was stained with acetocarmine to determine the fertility of the 0B, 1B, and 2B plants (**S6 Appendix**). The proportions of well-stained pollen (mature pollen grains) were 98.4% ± 0.7% (n = 3458) for 0B plants, 96.3% ± 0.1% (n = 2728) for 1B plants, and 96.0% ± 0.7% (n = 2131) for 2B plants. Pollen grains of *A*. *lancea* are trinucleate pollen and, therefore, contain three haploid nuclei (i.e., each contains one pollen tube nucleus and two sperm nuclei). No morphological abnormalities among these nuclei were detected in the pollen of 1B plants compared to that of 0B plants (**S6 Appendix**).

### Male transmission rates of B chromosomes

**Fig 7:**
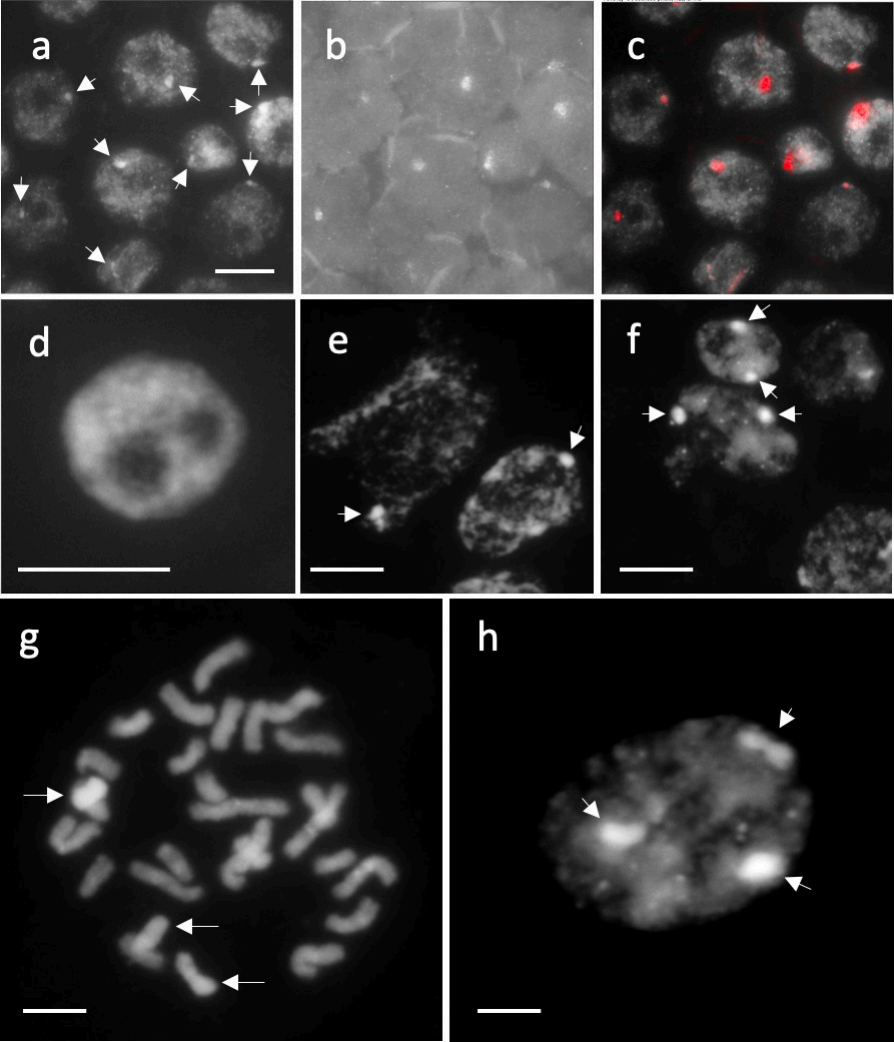
Transmission of B chromosomes to progeny. (a–c) FISH with B probe (probe #9) to the somatic cells of the progeny from a KY17-19 cross (1B; 2*n* = 25. See S1 Table). (a) DAPI-stained nuclei. Arrows indicate B-DAPI-bright regions. (b) FISH signal for the B probe. (c) Merged image. DAPI-bright signals overlapped with the B-specific signals. (d) Nucleus without B-DAPI-bright regions (0B). (e) Nuclei carrying a B-DAPI-bright region (arrows) (1B). (f) Nuclei carrying two B DAPI-bright regions (arrows) (2B). (g–h) Progeny (2*n* = 24 + 3Bs) from the KY17-29 (2*n* = 26) × KY17-22 (2*n* = 24) cross. (a) Mitotic chromosomes. Arrows indicate three Bs. (b) Nucleus. Arrows indicate three DAPI-bright regions derived from three Bs. Scale bar = 10 μm.

In the 1B plants, the B chromosome is transmitted to 18.6%–20.1% of the microspores. Moreover, pollen containing B chromosomes was found not to show morphological abnormalities. If genetic drive did not occur, pollen with the B chromosome should be transmitted to subsequent generations at the same frequency. We, therefore, conducted several cross-combinations to verify whether pollen with B could be transmitted to subsequent generations. We here collected data on the transmission rate of B chromosomes to the next generation by counting the DAPI-bright regions derived from the B chromosome (**Tables 3 and S2 and Fig 7A–7F**). Before this analysis, the B-specific probes confirmed that the DAPI-bright regions were derived from the B chromosomes in the 1B line, KY17-19 (**Fig 7A–7C and S1 Table**).

**Table 3:** Number of B chromosome in progenies from 14 cross combinations.

Cross combination (female × male)		Obs. ^1)^	No. of progenies (%)				*k*_*B*_	Z ^2)^
			0B	1B	2B	3B		
0B × 1B	KY17-21 × KY17-148	250	148 (59.2%)	102 (40.8%)	-	-	0.408	**-2.909**
	KY17-5 × KY17-15	33	21 (63.6%)	12 (36.4%)	-	-	0.364	-1.567
	KY17-45 × KY17-118	53	28 (52.8%)	25 (47.2%)	-	-	0.472	-0.412
	KY17-45 × KY17-148	52	29 (55.8%)	23 (44.2%)	-	-	0.442	-0.832
0B × 2B	KY17-43 × YB2019-3	47	6 (12.8%)	29 (61.7%)	12 (25.5%)	-	0.564	0.875
	KY17-5 × YB2019-3	98	23 (23.5%)	60 (61.2%)	15 (15.3%)	-	0.459	-0.808
1B × 0B	KY17-7 × KY17-60	11	2 (18.2%)	9 (81.8%)	-	-	0.818	**2.111**
	KY17-30 × KY17-37	10	3 (30.0%)	7 (70.0%)	-	-	0.700	1.265
	KY17-6 × KY17-22	81	6 (7.4%)	75 (92.6%)	-	-	0.926	**7.667**
	KY17-38 × KY17-22	98	35 (35.7%)	63 (64.3%)	-	-	0.643	**2.828**
2B × 0B	KY17-29 × KY17-22	77	14 (18.2%)	24 (31.2%)	38 (49.4%)	1 (1.2%)	0.669	**2.963**
	YT16-69 × KY17-60	14	0 (0.0%)	7 (50.0%)	7 (50.0%)	-	0.750	1.871
1B × 2B	KY17-30 × YB2019-3	51	3 (5.9%)	31 (60.8%)	17 (33.3%)	0 (0.0%)	0.425	-1.074
2B × 1B	YT16-69 × KY17-148	122	5 (4.1%)	40 (32.8%)	60 (49.2%)	17 (13.9%)	0.577	0.003

^1)^ Number of observed progenies.

^2)^ B accumulation

(Z values higher than 1.96) and B elimination (Z values lower than -1.96) were indicated by underline.

In the cross of 0B females and 1B males, 36.4%–47.2% of the progeny contained a B chromosome (1B), and the remaining 63.6%–52.8% did not (0B). One (KY17-148) of three males analyzed demonstrated B elimination (*k*_*B*_ = 0.408; Z = –2.909) in a cross with a KY17-21 female; it also showed Mendelian transmission (*k*_*B*_ = 0.442; Z = –0.832) in a cross with a KY17-45 female. Two other males (KY17-15 and KY17-118) also demonstrated Mendelian transmission (**Table 3**). Moreover, the transmission rates (40.8%–44.2% and 47.2%, respectively) of B chromosomes from KY17-148 (1B) and KY17-118 (1B) males were more than twice those (i.e., 18.6%–20.1%) of microspores containing B chromosomes in these lines (**Table 2**). Finally, a cross involving two 0B females (KY17-43 and KY17-5) showed that one 2B male (YB2019-3) transmitted its B chromosome at the Mendelian ratio (**Table 3**).

### Female transmission rates of B chromosomes

Due to technical difficulties, we could not observe female meiosis inside the pistil. If the 1B lines were used as the female parent for crosses under the non-drive hypothesis, the B chromosome would be transmitted to 50% of the progeny. We analyzed the transmission of B chromosomes from the female side to the subsequent generations using the lines with B chromosomes for the crosses.

In three of four cross combinations involving four 1B females and three 0B males, B chromosomes were transmitted to 64.3%–92.6% of all progeny, which suggests B chromosome accumulation (*k*_*B*_ = 0.818, 0.926, and 0.643 and Z = 2.111, 7.667, and 2.828, respectively) (**Table 3**). The remaining cross combination with KY17-30 demonstrated Mendelian transmission (*k*_*B*_ = 0.700; Z = 1.265), although this result may be related to the small number of progenies analyzed (**Table 3**). For the cross between the 2B female (KY17-29 and YT16-69) and 0B male, two 2B females exhibited B chromosome accumulation; however, *k*_*B*_ was not significantly different from 0.5 in the cross between YT16-69 and KY17-60 (**Table 3**). We also found one plant (1.2% of the total) from the cross between KY17-29 (2B) and KY17-22 (0B) that showed 2*n* = 27 (3B) in the cross between KY17-29 (2B) and KY17-22 (0B) (**Table 3 and Fig 7G and 7H**). Moreover, 14 progenies (18.2%) from the cross combination did not carry Bs (**Table 3**); if the two Bs were paired and segregated during meiosis, 0B would not appear.

### Transmission of B chromosomes in 2022 and 2023 and crossings between B parents

Next, we tested whether the environment affected the B transmission rates. A cross between KY17-21 (0B) and KY17-148 (1B) and a cross between KY17-6 (1B) and KY17-60 (0B) were conducted in both 2022 and 2023. We found that the cross between KY17-21 (0B) and KY17-148 (1B) demonstrated B chromosome elimination in 2022 but showed a Mendelian ratio in 2023 (**S2 Table**). In contrast, the cross between KY17-6 (1B) and KY17-60 (0B) demonstrated B chromosome accumulation in both 2022 and 2023 (**S2 Table**).

Finally, we performed the 1B × 2B and 2B × 1B crosses. For the 1B × 2B and 2B × 1B crosses to investigate the B transmission rates between B chromosome-containing parents. Bs were transmitted in Mendelian ratios (**Table 3**). In addition, similar to that in the cross between KY17-29 (2B) and KY17-22 (0B), we also found 0B progenies (5.9% in 1B × 2B and 4.1% in 2B × 1B) in the following crosses (**Table 3**).

## Discussion

### Structure of *A*. *lancea* B chromosomes

Polymorphisms in the number and morphology of chromosomes can arise from polyploidy, aneuploidy, and chromosomal rearrangements. The extra chromosomes of *A*. *lancea* detected in this study were determined to be novel B chromosomes because they exhibited no noticeable effect on plant morphology and vitality (**Fig 1**), did not pair with an A chromosome (**Fig 4**), and displayed non-Mendelian inheritance from female parents (**Table 3**). We considered the possibility that they are a sex chromosome present in this gynodioecious plant, but we found that the extra chromosome was not a direct sex determinant (**Tables 1 and S1**).

Our FISH analyses also indicated that the B chromosomes of *A*. *lancea* did not contain rDNA (**S2 Appendix**). Based on the B-specific sequences and the sharing of similar DNA in the centromeric region as revealed by the GISH analysis (**Fig 3**), we inferred two possible evolutionary scenarios: intraspecific construction and hybrid origin. When neo-Bs appear intraspecifically, they can undergo a rapid structural modification that inhibits meiotic pairing with homologous progenitors. Among the various models of B chromosome origin, a report suggests that B chromosomes evolve due to the amplification of B-specific repeats from centromeres or small chromosome fragments [44]. For example, a fragment of the *A*. *lancea* centromere that shares a sequence with an A chromosome contains rapidly accumulated B-specific repeats to favor stable meiosis and drive, leading to the formation of the present B chromosome. Although we did not determine the DNA sequences specific to *A*. *lancea* B chromosomes in this study, the constitutive DAPI-bright region resembling heterochromatin suggests the presence of repetitive sequences throughout the chromosome arm. Furthermore, the number of B chromosomes was the same in tissue samples taken from both the root tips and anthers of *A*. *lancea*, and Bs were observed to be stable during somatic cell division. Hence, the *A*. *lancea* centromere, which acts as the primary constriction and sequence conservation, may also stabilize Bs during somatic cell division. Moreover, this drive may involve newly acquired B sequences rather than differences in the centromere. In rye, the distal region of the long arm of the B chromosome was found to be required for nondisjunction [13]. In the second scenario, Bs are formed from the A chromosomes of closely related species [45]. This hybrid origin of Bs can be detected by the presence of B repeats from closely related species, as has been found in the wasp *Nasonia* [46]. Moreover, the growth areas of the closely related species *Atractylodes chinensis* and *Atractylodes japonica* [47] overlap with that of *A*. *lancea*, and hybrids between these species exist in natural populations. Nevertheless, there is a lack of sequence information for *A*. *lancea* Bs, and no evidence from the *A*. *chinensis* and *A*. *japonica* karyotypes supports the hybrid-origin scenario.

Sequencing of Bs is a definitive approach to understanding their structure and evolution. Microdissection and cloning of the Bs of *Brachycome dichromosomatica* revealed that the micro B chromosome consists of two tandem repeats (i.e., Bdm29 and Bdm54) that are not present in the A chromosome, indicating that the macro B chromosome was not established by simple A chromosome excision [24]. In rye, the B chromosome was isolated by chromosome flow sorting; its shotgun sequencing revealed that the Bs were primarily derived from the rye chromosomes 3R and 7R [48]. In another study, the sequence of the 570-Mbp *Ae*. *speltoides* B chromosome was determined by comparative sequence analysis and B microdissection [22]. In still another, a high-quality DNA sequence of maize Bs derived from a combination of chromosome flow sorting, Illumina sequencing, Bionano, and Hi-C analyses was determined, and the assembly revealed that the maize B is gene-rich due to continuous transfer from the A chromosome [25]. In this study, we found that the Bs of *A*. *lancea* are relatively large and display DAPI-bright signals that distinguish them from A chromosomes. Interestingly, this B chromosome could be observed as a DAPI-bright region in the nucleus, consistently showing the same size and distinct primary constriction. The observation may indicate that this B remains condensed in the nucleus for most of the cell cycle or is preferentially stained with DAPI to a greater degree than A chromosomes. These structural features helped conduct microdissection in this study and may be advantageous for chromosome flow sorting, which is required for sequencing analysis.

### Non-Mendelian inheritance of Bs in *A*. *lancea*

**Fig 8:**
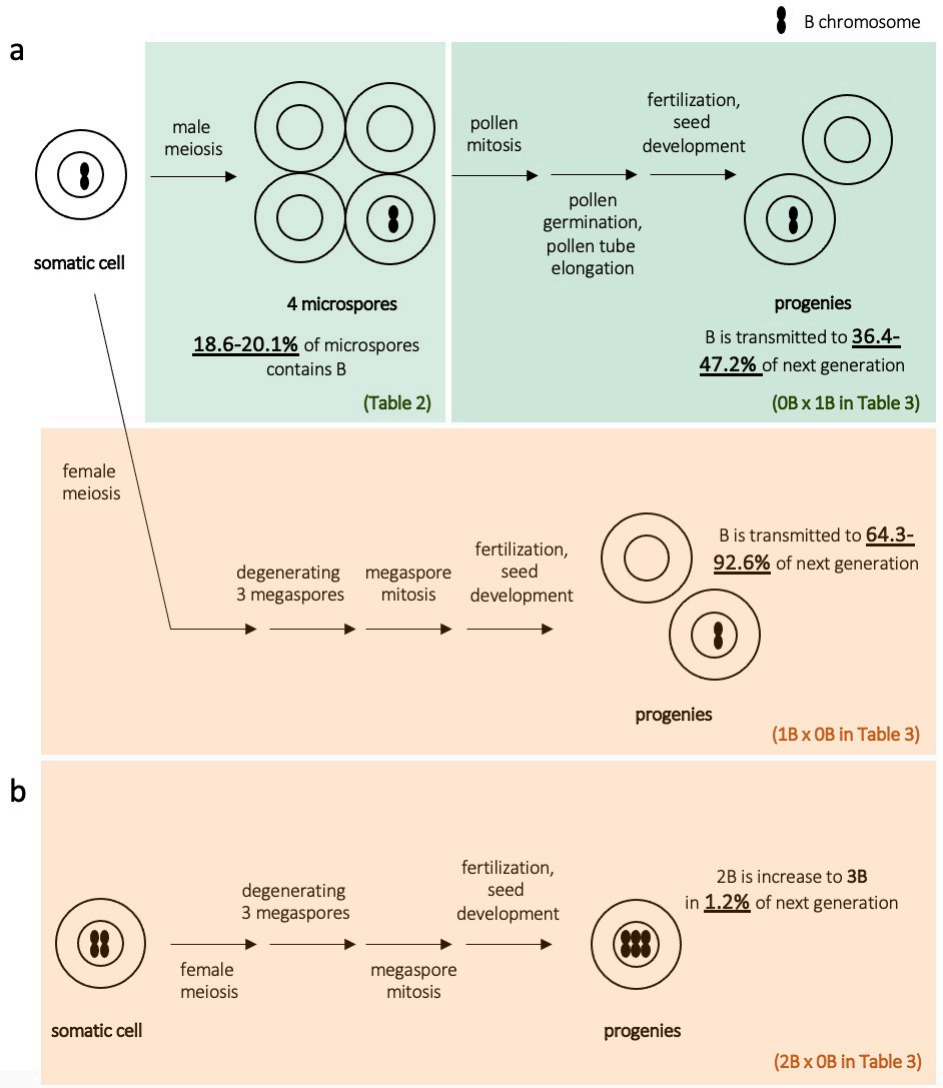
Summary of the transmission of the *A*. *lancea* B chromosome. (a) In the 1B line, univalent B was transmitted to 18.6%–20.1% of microspores in male meiosis due to chromosome elimination. In contrast, the B chromosome was transmitted to 36.4%–47.2% of cross progeny, suggesting preferential fertilization during pollen mitosis, the germination of pollen grains, or pollen tube growth. In crosses where the 1B plant is female, the B chromosome was transmitted to 64.3%–92.6% of progeny, suggesting female drive or transmission drive during meiosis, megaspore degeneration, fertilization, or seed development. (b) One progeny containing three B chromosomes was found in the 2B × 0B cross, suggesting that nondisjunction occurs during female meiosis, following mitosis (megasporogenesis), fertilization, or seed development.

The nature of B transmission observed in *A*. *lancea* is summarized in **Fig 8**. During male meiosis, the Bs of *A*. *lancea* plants with 1B formed an univalent chromosome. They were transmitted to 18.6%–20.1% of the microspores (i.e., the daughter cells produced by male meiosis), suggesting the loss of univalent B (**Fig 6**) such as interphase elimination or passive B chromosome loss during chromatid segregation [49, 50] without a drive mechanism. In a report studying programmed elimination in *Ae*. *speltoides*, B chromosomes with active centromere lagged in anaphase and formed micronuclei [22]. Generally, a univalent chromosome is easy to eliminate from meiosis; for instance, the transmission rate was reported to be as low as 25% in monosomic wheat series [51, 52]. However, the maize *B-9* univalent can reach one pole ahead of A chromosomes [53], and this chromosome did not show the regular number of centromere repeats [54, 55]. Although Bs can maintain or even increase their number in male meiosis in other plant and animal species [56, 57], our results suggest that no such male meiotic drive probably exists for *A*. *lancea*. Since almost all the microspores became mature pollen (**S6 Appendix**), the B chromosome was expected to be transmitted to 18.6%–20.1% of the pollen and the same proportion of the next generation. However, it was transmitted to 40.8%–47.2% of the next generation (**Table 3**). Except for the cross KY17-21 × KY17-148, the transmission rate from male parents did not significantly differ from Mendelian inheritance (0.5) (**Table 3**). However, it was approximately twice the rate observed for microspores (i.e., 18.6%–20.1%). Moreover, we observed no variation in the number of Bs per cell, as in the case of the nondisjunction of rye and maize B chromosomes. We also detected no abnormalities in the germination of mature seeds or plant growth. Therefore, we speculate that an unknown mechanism involved in preferential reproduction (i.e., fertilization and seed development) of pollen-carrying Bs may cause a higher rate of B transmission. In rye, *in vitro* tests of pollen grain germination and pollen tube growth show that pollen from plants carrying Bs exhibits better performance than pollen produced from plants without Bs [58]. A previous study has also reported on the effect of Bs on development in grasshopper embryos [59]. The B-specific probes developed in this study may be helpful for further analysis of the mechanistic reason for the higher rate of B transmission in pollen mitosis, germination of pollen grains, pollen tube growth, and fertilization in *A*. *lancea*.

Unlike the transmission from male parents, maternal univalent B chromosomes were transmitted to 64.3%–92.6% of progenies, and B chromosome accumulation was significantly present in at least three of four cross combinations (**Fig 8 and Table 3**). Although we have no cytological evidence, female drive may explain the higher transmission rate of *A*. *lancea* Bs. The higher transmission rate is because the asymmetries in female meiosis and meiotic daughter cell (megaspore) formation provide an opportunity for meiotic drive. One of the four megaspores develops into an egg cell, whereas the remaining three are not involved in reproduction. Bs may cause female meiotic drive and are preferentially transmitted to the nucleus of cells that will become egg cells, resulting in a transmission rate >0.5. During female meiosis in *Lilium callosum*, one univalent B chromosome has been found to lie on the micropylar side of the equatorial plate and is transmitted to 80% of the next generation [21]. Furthermore, in rye and *Ae*. *speltoides*, extensive observations suggest that the asymmetric formation of microtubules induces nondisjunction rather than a dysfunctional centromere [11, 14, 60]. Interestingly, a recent study of Drosophila by Hanlon and Hawley [61] showed that the female B transmission to progeny in a specific genetic background is non-drive. However, in a mutant background, the authors observed a biased transmission of the B chromosomes on the female side, thereby indicating a meiotic drive suppression system.

Like the 1B × 0B cross, *A*. *lancea* 2B female plants also tended to preferentially transmit Bs to the next generation (**Table 3**). However, if two B chromosomes pair during meiosis, the B chromosome should be transmitted at a rate of 100%, which is inconsistent with the appearance of 0B progeny (i.e., 18.2% from KY17-29 × KY17-22 cross, 5.9% from KY17-30 × YB2019-3 cross, and 4.1% from YT16-69 × KY17-148 cross). We also found 2*n* = 27 progeny (1.2%) in a 2B × 0B *A*. *lancea* plant cross (**Table 3**). The mechanism of B chromosome transmission, including those responsible for the rare cases listed above, needs to be clarified by future cytological analyses. In a cross between 2B and 1B, Bs were transmitted according to the Mendelian ratio, possibly due to the progeny of the transmission ratio of B accumulation on the female side and weak B elimination on the male side (although the transmission ratios from most male parents are not significantly different from Mendelian transmission). Alternatively, since no >3B lines were observed among the 54 lines analyzed, the accumulation of the B chromosome might be harmful in *A*. *lancea*. However, the 2B lines used in this study did not show weaknesses relative to 0B and 1B lines.

## Conclusions

In this paper, we report the identification of a new B chromosome in *A*. *lancea*. Subsequently, we characterized its structure and the biased B transmissions. The Bs appeared as a DAPI-bright region in the nuclei, suggesting an unusual chromatin condensation pattern or preferential staining with DAPI. On the male side, Bs are lost during micronucleation or chromatid segregation, but there seems to be a mechanism by which gametes with Bs are preferentially fertilized. On the female side, a relatively strong drive was found; this transmitted Bs to ~92.6% of the progeny. The biased B transmission pattern among male and female parents is unique and may provide new insights into B chromosome biology.

## Supporting information

S1 Appendix Arm ratios of B_1_ and B_2_ in Giemsa−stained chromosome images. (A) B_1_ and B_2_ stained with Giemsa solution. Scale bar = 10 μm. (b) Y-axes show arm ratios (L/S: long arm / short arm). A statistically significant difference (P < 0.01) was detected for the arm ratios of B_1_ and B_2_.
(TIF)

S2 Appendix 35S rDNA-FISH of *A*. *lancea* line Y-T16-69 (2*n* = 26, 2B). (a) DAPI-stained mitotic chromosomes. (b) The FISH image. The B chromosomes (i.e., B_1_ and B_2_, indicated by yellow arrows) do not contain rDNA. Red arrows indicate the four loci of 35S rDNA. The white arrow indicates a non-specific signal. Scale bar = 10 μm.
(TIF)

S3 Appendix DAPI-bright univalent in meiotic metaphase I. (a) Three DAPI-stained meiotic metaphase I cells. Univalent chromosomes (yellow arrows) are observed in each cell. (b–d) Close-up images of the three metaphase I cells. Scale bars = 10 μm.
(TIF)

S4 Appendix Microspore after male meiosis in KY17-15 (1B). (a) The nucleus of the microspore does not show a DAPI-bright region. (b) The nucleus of the microspore contains a DAPI-bright region. Scale bar = 10 μm.
(TIF)

S5 Appendix FISH analysis probed with three dissected DNA samples (i.e., #8, 5, 2). All three probes hybridize specifically to B chromosomes (arrows). Scale bars = 10 μm.
(TIF)

S6 Appendix Acetocarmine staining of pollen grains. (a) KY17-60 (0B). The cytoplasm of most pollen grains was stained; the arrow indicates an unstained grain. (b) KY17-15 (1B). (c, d) Two generative nuclei and a vegetative (pollen tube) nucleus (c) KY17-60 (0B) and (d) KY17-15 (1B). No morphological differences were observed between the nuclei (c) and (d). Scale bars = 10 μm.
(TIF)

S1 Table (PDF)

S2 Table (PDF)

## Abbreviations

Bs: B chromosomes
FISH: fluorescence *in situ* hybridization
GISH: genomic *in situ* hybridization
NORs: nucleolar organizer regions
rDNA: ribosomal DNA
