# Supplementary figures and images for: B chromosome and its non-Mendelian inheritance in Atractylodes lancea
Source: PLoS One. 2024 Sep 11;19(9):e0308881. doi: 10.1371/journal.pone.0308881 (PMC11389924; doi:10.1371/journal.pone.0308881)

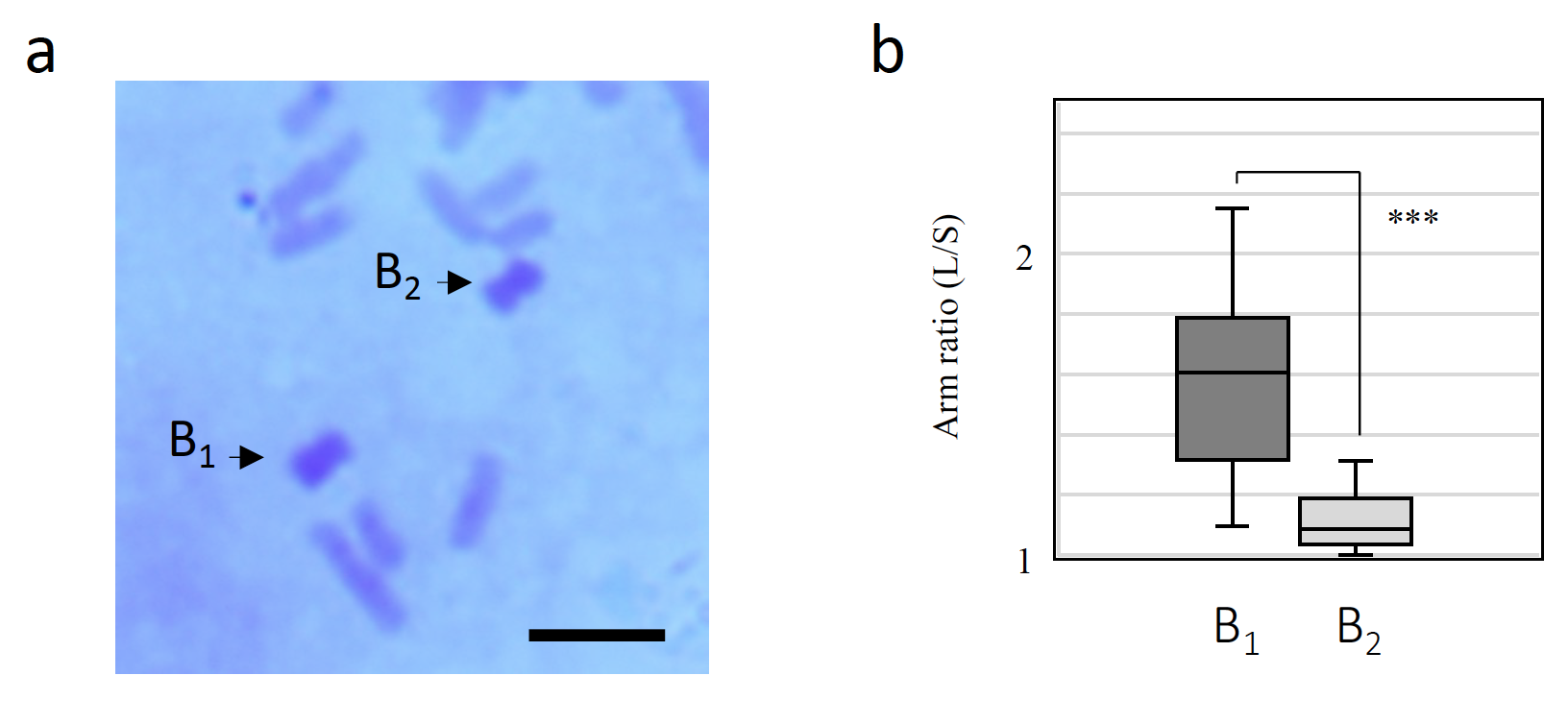

Supplement: S1 Appendix — (A) B1 and B2 stained with Giemsa solution. Scale bar = 10 μm. (b) Y-axes show arm ratios (L/S: long arm / short arm). A statistically significant difference (P < 0.01) was detected for the arm ratios of B1 and B2. (TIF) [file pone.0308881.s001.tif]

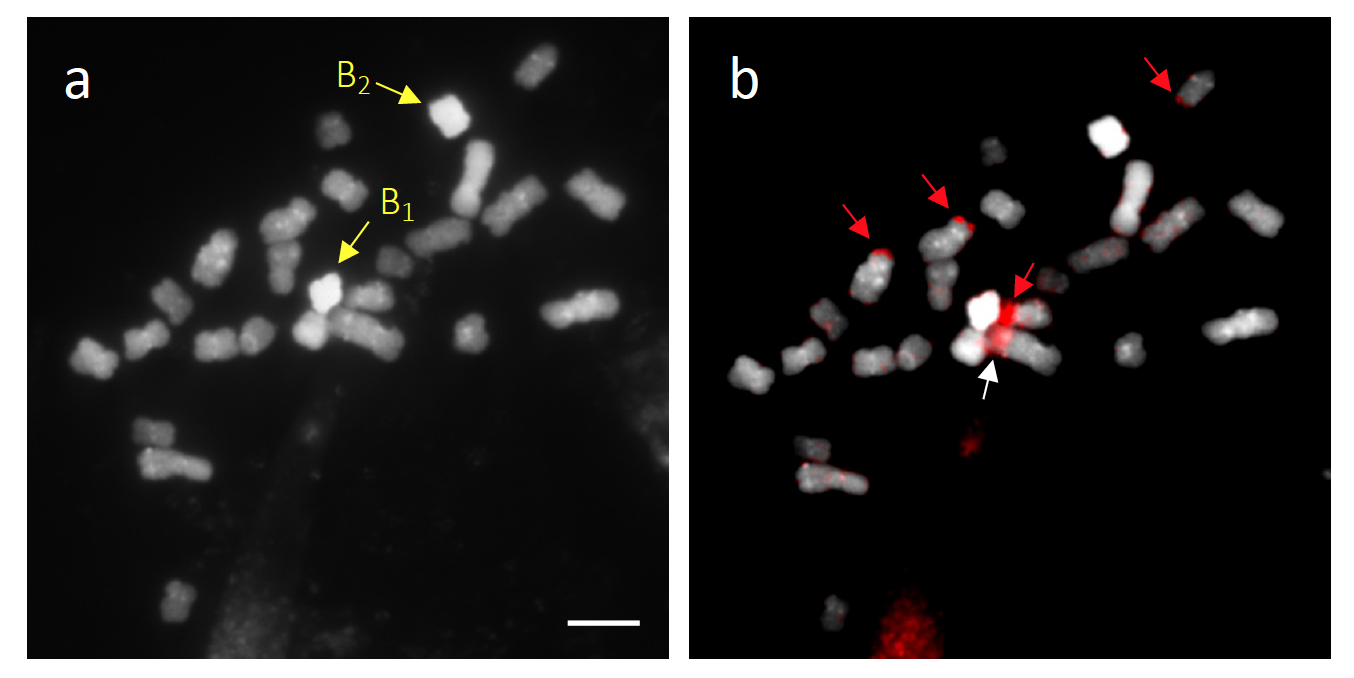

Supplement: S2 Appendix — (a) DAPI-stained mitotic chromosomes. (b) The FISH image. The B chromosomes (i.e., B1 and B2, indicated by yellow arrows) do not contain rDNA. Red arrows indicate the four loci of 35S rDNA. The white arrow indicates a non-specific signal. Scale bar = 10 μm. (TIF) [file pone.0308881.s002.tif]

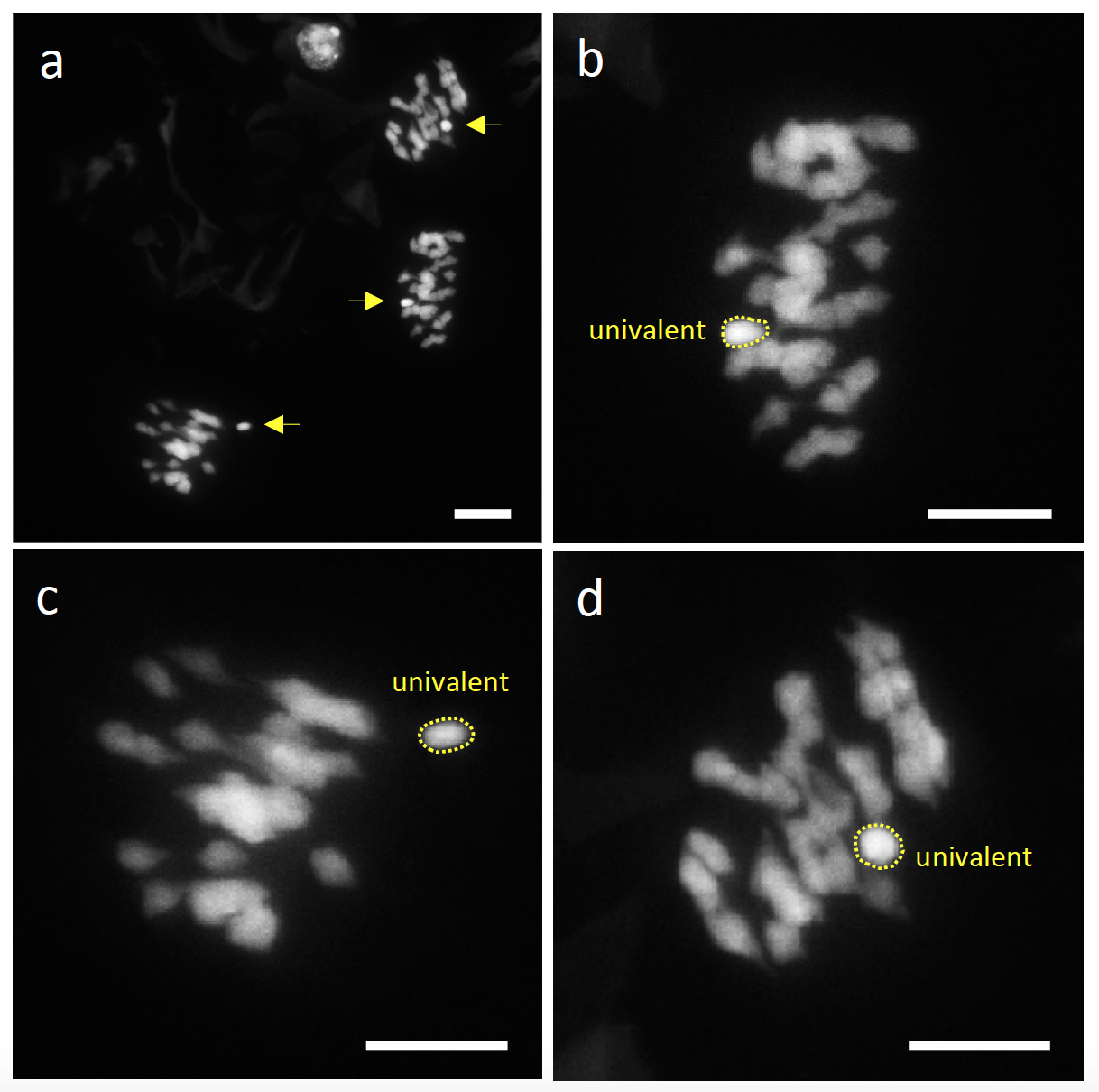

Supplement: S3 Appendix — (a) Three DAPI-stained meiotic metaphase I cells. Univalent chromosomes (yellow arrows) are observed in each cell. (b–d) Close-up images of the three metaphase I cells. Scale bars = 10 μm. (TIF) [file pone.0308881.s003.tif]

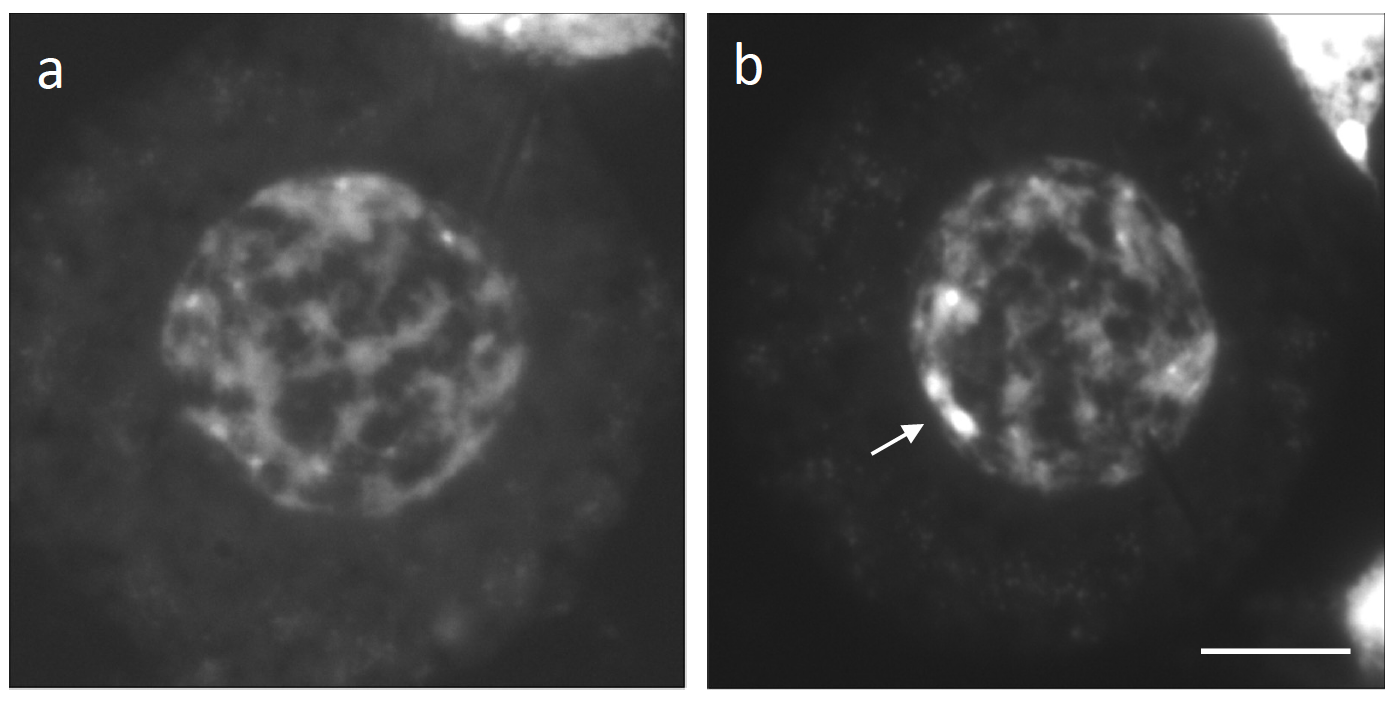

Supplement: S4 Appendix — (a) The nucleus of the microspore does not show a DAPI-bright region. (b) The nucleus of the microspore contains a DAPI-bright region. Scale bar = 10 μm. (TIF) [file pone.0308881.s004.tif]

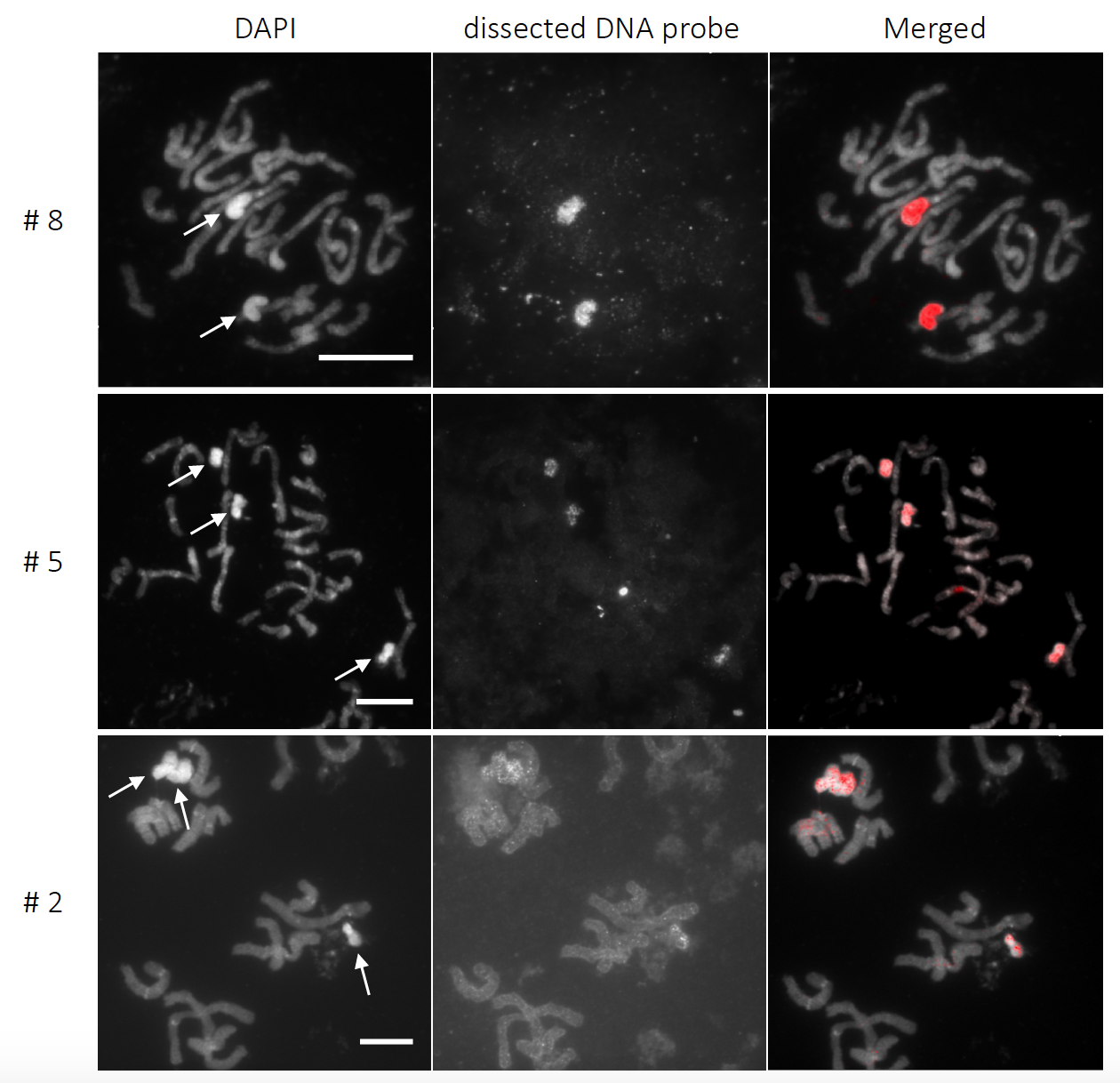

Supplement: S5 Appendix — All three probes hybridize specifically to B chromosomes (arrows). Scale bars = 10 μm. (TIF) [file pone.0308881.s005.tif]

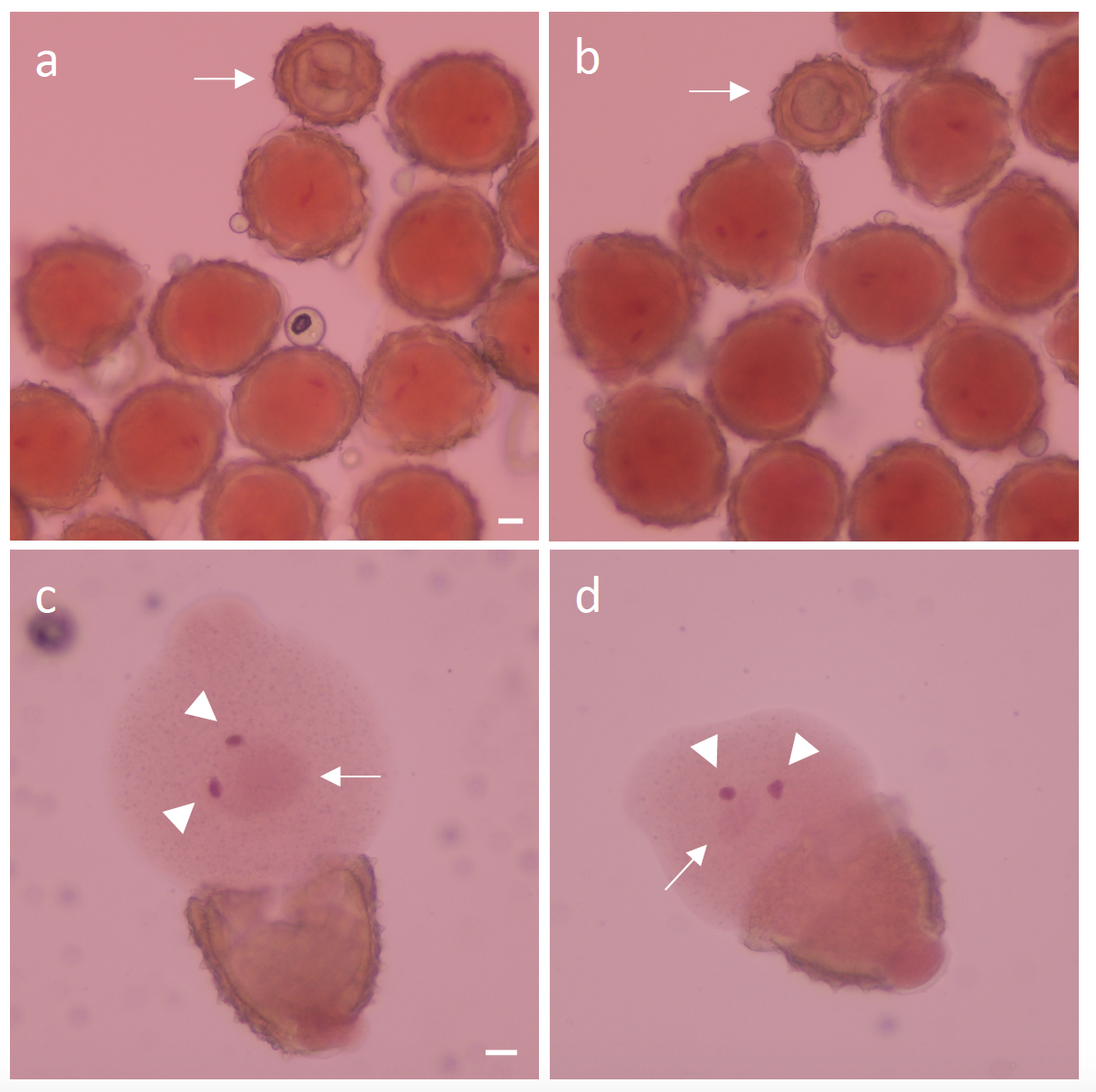

Supplement: S6 Appendix — (a) KY17-60 (0B). The cytoplasm of most pollen grains was stained; the arrow indicates an unstained grain. (b) KY17-15 (1B). (c, d) Two generative nuclei and a vegetative (pollen tube) nucleus (c) KY17-60 (0B) and (d) KY17-15 (1B). No morphological differences were observed between the nuclei (c) and (d). Scale bars = 10 μm. (TIF) [file pone.0308881.s006.tif]
