# Supplementary material for: B chromosome and its non-Mendelian inheritance in Atractylodes lancea
Source: PLoS One. 2024 Sep 11;19(9):e0308881. doi: 10.1371/journal.pone.0308881 (PMC11389924; doi:10.1371/journal.pone.0308881)
Supplement: S1 Table — (PDF) [file pone.0308881.s007.pdf]

S1 Table. Chromosome number (2*n*) and B chromosome in 54 *A. lancea* lines.

Line ID	Sex phenotype	2*n*	No. of B chr. [1)]
Y-T16-69	female	26	2
5-7-32	female	24	0
5-2-12	female	24	0
5-12-7	female	24	0
KY17-4	female	24	0
KY17-5	female	24	0
KY17-6	female	25	1
KY17-7	female	25	1
KY17-8	female	24	0
KY17-10	hermaphroditic	25	1
KY17-15	hermaphroditic	25	1
KY17-16	female	25	1
KY17-19	female	25	1
KY17-21	female	24	0
KY17-22	hermaphroditic	24	0
KY17-28	hermaphroditic	24	0
KY17-29	female	26	2
KY17-30	female	25	1
KY17-34	female	24	0
KY17-37	hermaphroditic	24	0
KY17-38	female	25	1
KY17-41	female	25	1
KY17-43	female	24	0
KY17-45	female	24	0
KY17-47	hermaphroditic	24	0
KY17-48	hermaphroditic	24	0
KY17-51	female	24	0
KY17-57	hermaphroditic	24	0
KY17-59	female	25	1
KY17-60	hermaphroditic	24	0
KY17-61	female	24	0

KY17-62	female	25	1
KY17-69	female	24	0
KY17-75	hermaphroditic	24	0
KY17-84	female	25	1
KY17-93	hermaphroditic	24	0
KY17-100	female	25	1
KY17-113	female	24	0
KY17-117	hermaphroditic	24	0
KY17-118	hermaphroditic	25	1
KY17-122	female	24	0
KY17-143	hermaphroditic	24	0
KY17-146	hermaphroditic	24	0
KY17-148	hermaphroditic	25	1
KY17-151	hermaphroditic	24	0
KY17-171	hermaphroditic	24	0
YB2019-3	hermaphroditic	26	2
YB2019-5	hermaphroditic	24	0
YB2019-30	hermaphroditic	25	1
YB2019-34	hermaphroditic	24	0
YB2019-35	hermaphroditic	25	1
YB201937	hermaphroditic	24	0
YB2019-54	hermaphroditic	24	0
YB2019-119	hermaphroditic	25	1

[1)] Lines carrying B chromosome(s) are highlighted.
