# Supplementary material for: B chromosome and its non-Mendelian inheritance in Atractylodes lancea
Source: PLoS One. 2024 Sep 11;19(9):e0308881. doi: 10.1371/journal.pone.0308881 (PMC11389924; doi:10.1371/journal.pone.0308881)
Supplement: S2 Table — (PDF) [file pone.0308881.s008.pdf]

S2 Table. Number of B chromosome in progenies from 2 cross combinations in 2022 and 2023.

Female × Male	Cross combination	Year	Obs.	No. of progenies (%)				k_B	Z [1)]
				0B	1B	2B	3B		
0 B × 1 B	KY17-21 × KY17-148	2022	147	91 (61.9%)	56 (38.1%)	-	-	0.381	**-2.887**
	KY17-21 × KY17-148	2023	103	57 (55.3%)	46 (44.7%)	-	-	0.447	-1.084
1 B × 0 B	KY17-6 × KY17-22	2022	32	3 (9.4%)	29 (90.6%)	-	-	0.906	**4.596**
	KY17-6 × KY17-22	2023	49	3 (6.1%)	46 (93.9%)	-	-	0.939	**6.143**

[1)] B accumulation (Z values higher than 1.96) and B elimination (Z values lower than -1.96) were indicated by underline.
